# Berberine for Appetite Suppressant and Prevention of Obesity

**DOI:** 10.1155/2020/3891806

**Published:** 2020-12-12

**Authors:** Hyun-Jung Park, EunYee Jung, Insop Shim

**Affiliations:** ^1^Department of Food Science & Biotechnology, College of Science and Engineering, Kyonggi University, 154-42, Gwanggyosan-ro, Yeongtong-gu, Suwon-si, Gyeonggi-do 16227, Republic of Korea; ^2^Department of Physicology, College of Medicine, Kyung Hee University, Seoul 130-701, Republic of Korea

## Abstract

Berberine (BBR), a natural plant product, has been shown to have antidiabetic, cholesterol-reducing effects. To investigate the action of BBR as appetite suppressants, two experimental protocols were performed. In the first experiment, the mice were fed either a normal-chow diet or a high-fat diet (HF). The mice received daily intraperitoneal injections of BBR (10 mg/kg or saline at 1 ml/kg) for 3 weeks. To determine the antiobesity effects of BBR, the food consumption, body weight, fat contents, serum leptin, and glucose level were investigated. In the second experiment, we set out to validate the effect of BBR on central neuropeptide Y (NPY) stimulated rats. Experiments were carried out in 24-hour fasted rats, and then food intake and glucose level were subsequently recorded for 1 hour. The experimental groups were subdivided into the intra-3rd ventricular microinjections of ACSF (artificial cerebrospinal fluid), neuropeptide Y (NPY; 100 nM), NPY+BBR (10 nM), and NPY+BBR (100 nM) group. And then the blood glucose level was examined. In the first experiment, treatment with BBR in the HF diet mice reduced food intake, body weight, fat contents, serum leptin, and glucose level. In the second experiment, the NPY-injected group increased food intake by 39.3%, and food intake was reduced in the BBR group by 47.5%, compared with the ACSF-injected group. Also, the serum glucose level in the NPY+BBR (100 nM) group was significantly lower than that in the NPY (100 nM) group. The results suggest that BBR improved lipid dysregulation in obesity by controlling the central obesity related pathway.

## 1. Introduction

Recently, epidemiological evidences have shown a positive relationship between obesity and dietary fat intake. Many studies reported that rats and mice prove a similar relationship, and they showed an appropriate model for dietary obesity studying [1–3]. Overweight and obesity are related as excessive fat accumulation or abnormal that presents a risk to health. An overview of animal studies had shown that increased fat intake is associated with metabolic disease and cardiovascular-related diseases.

Obesity is defined medically as a state of increased body weight [4] and is associated with several medical problems such as cardiovascular disease, type 2 diabetes, and several cancers [5]. Body is need for energy which is obtained from food. If energy intake exceeds total body energy expenditure, stored energy increases. Thus, increased food intake explains the increase in body weight vice versa. During the last few decades, many studies have shown that berberine has various beneficial effects on the antimicrobial activity [6–8], treatment of trachoma [9], anti-inflammatory activity [10], and type 2 diabetes [11]. Berberine inhibits adipogenesis in high-fat diet-induced obesity mice [12] and 3T3-L1 adipocyte differentiation through the PPAR gamma pathway [13]. Although berberine was demonstrated to have antiadipogenic effects *in vitro*, its overall performance *in vivo* was poor.

To investigate the action of BBR as appetite suppressants, two experimental protocols were performed. In the first experiment, the mice were fed either a normal-chow diet or a high-fat diet (HF). The mice received daily intraperitoneal injections of BBR (10 mg/kg or saline at 1 ml/kg) for 3 weeks. To determine the antiobesity effects of BBR, the food consumption, body weight, fat contents, serum leptin, and glucose level were investigated. In the second experiment, we set out to validate the effect of BBR on central neuropeptide Y (NPY) stimulated rats. Experiments were carried out in 24-hour fasted rats, and then food intake and glucose level were subsequently recorded for 1 hour. The experimental groups were subdivided into the intra3rd ventricular microinjections of ACSF (artificial cerebrospinal fluid), neuropeptide Y (NPY; 100nM), NPY+BBR (10nM), and NPY+BBR (100nM) group. And then the blood glucose level was examined.

## 2. Methods and Materials

### 2.1. Experiment 1

#### 2.1.1. Animals and Diets

Four-week male C57BL/6 J mice (Orient Animal Corp, Kyunggido, Korea) with a body weight of 17.7 ± 1 g were used for the experiment. The mice were fed commercial mice chow for 1 week before they switched to an HF diet containing lard and cholesterol for 3 weeks. For the 3 weeks, the mice received daily intraperitoneal injections of berberine 10 mg/kg or saline at 1 ml/kg. The mice were maintained in a temperature-controlled room (18–26°C, 30%-70% relative humidity) with a 12 : 12 light-dark cycle and given free access to food and tap water. The experimental diets contained either normal fat (11.7% of calories as fat, AIN-76A diet #100000, Dyets Inc., Bethlehem, PA, USA), or high fat (40% of calories as fat, ANI-76A diet #100496, Dyets Inc., Bethlehem, PA, USA, Table 1).

#### 2.1.2. Experimental Designs

The experiment was designed to investigate the effect BBR on the HF-induced model of obesity. The mice were fed either a normal diet (N diet group) or a high-fat diet (HF group). The HF group comprised mice that fed an HFD that received no BBR treatment.

#### 2.1.3. Measurement of Food Intake, Body Weight

Food intake and body weight were recorded twice per week. The food cups were removed at 8 : 00 am and returned to animals with fresh food at 5 : 00 pm. In each group, the mice were collected randomly and weighed.

#### 2.1.4. Enzyme-Linked Immunosorbent Assay (ELISA) and Weight of Regional Fat

Following 4-5 h feed deprivation of feed, blood was drawn from the heart under sodium pentobarbital anesthesia (60 mg/kg, i.p.) and centrifuged (3,000 g for 15 min at 4°C). Subsequently, the epididymal fat, perirenal fat, and peritoneal fat pads were immediately excised, weighed, and frozen in liquid N_2_. Serum and tissue samples were frozen at -70°C until used for the measurement of biochemical parameters. The serum glucose (R&D system Inc, CA, USA) was determined by using an enzyme-linked immunosorbent assay (ELISA) kit. All serum samples were analyzed in duplicate in one assay, and intra- and interassay variation was below 10%.

### 2.2. Experiment 2

#### 2.2.1. Animals

All experimental procedures performed on the animals were conducted with the approval of the Ethics Committee of the Kyung Hee University and in accordance with the US National Institutes of Health “Guide for the care and use laboratory animals” (NIH Publication No. 80-23, revised 1996). Sprague-Dawley male SD rats (Orient Animal Corp, Kyunggido, Korea) that weighted 220 ± 240 g each were used for the experiment. The rats were maintained in a temperature-controlled room (18-26°C, 30%-70% relative humidity) with a 12 : 12 light-dark cycle and given free access to food and tap water. The skull was firmly placed in a stereotaxic apparatus. The skull was firmly placed in the apparatus, and the scalp was shaved and cleaned with betadine. An incision was made through the skin and muscle to expose the skull, and the skin was then retracted. Guide cannulas, 22-gauage, were implanted with tip at the following coordinates (nm from bregma): (3^rd^ ventricle) AP: -0.60, ML: -0.00, DV: -6.40). The rats were allowed 7 days to recover from surgery before testing. All the employed coordinates were from the atlas of Paxinos and Watson [14].

Experiments were carried out in 24-hour fasted rats. Rats were divided into four groups: ACSF-injected group (ACSF), NPY- (100 nM) injected group (NPY) and berberine- (10nM or 100nM) injected group (BBR). Drugs were injected into the 3^rd^ ventricle using the microinfusion pump (5 ul/min for 2 min). And food intake was subsequently recorded for 1 hour. After behavior tests immediately, all animals were deeply anesthetized with sodium pentobarbital (80 mg/kg, i.p.), and then the rats were transcardially perfused with PBS and then chilled with 4% paraformaldehyde in phosphate buffer (pH 7.4). The brains were sectioned coronally (30 *μ*m) on a freezing microtome.

#### 2.2.2. Cresyl Violet Staining

**Figure 1:**
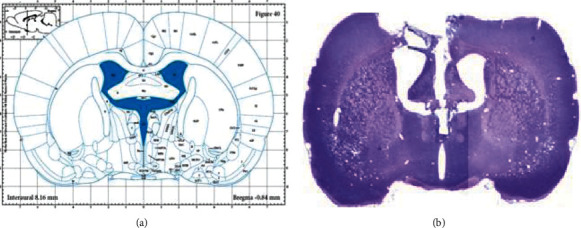
Location of the injection site in animals with cannulae aimed at the 3rd ventricle.

One set of sections that represented different regions of the 3rd ventricle was dehydrated, rehydrated, and stained with cresyl violet (ICN Biomedicals, Aurora, USA) to assess the morphology of the 3rd ventricle area (Figures 1(a) and 1(b)).

#### 2.2.3. Enzyme-Linked Immunosorbent Assay (ELISA)

After the behavior test, blood was drawn from the heart under sodium pentobarbital anesthesia (60 mg/kg, i.p.) and centrifuged (3, 000 g for 15 min at 4°C). Serum samples were frozen at -70°C until used for the measurement of biochemical parameters. The serum glucose (R&D system Inc, CA, USA) was determined by using an enzyme-linked immunosorbent assay (ELISA) kit. All serum samples were analyzed in duplicate in one assay, and intra- and interassay variation was below 10%.

#### 2.2.4. Statistical Analysis

The values of the experimental results were expressed as the mean ± S.E.M. Differences between groups were analyzed by analysis of variance (ANOVA) with or without repeated measures (time) as applicable. Individual comparisons among groups were analyzed by one-way ANOVA followed by Tukey's post hoc test. For all results, values of *P* < 0.05 were considered to indicate statistical significance. Analyses were computed by using SPSS statistical software (version 15.0 for Windows).

## 3. Results

### 3.1. Experiment 1

#### 3.1.1. Body Weight and Food Intake

**Figure 2:**
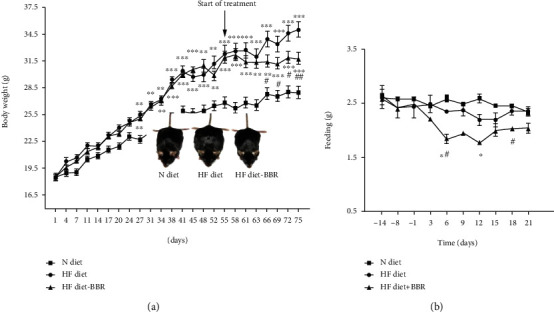
The change of body weight and food intake of the mice. ^∗^*P* < 0.05, ^∗∗^*P* < 0.01, ^∗∗∗^*P* < 0.001 N diet group vs. HF diet group. #*P* < 0.05, ##*P* < 0.01, *P* HF diet group vs. berberine group.

The change of body weight and food intake were shown in (Figures 2(a) and 2(b). Body weight was gradually increased with time but was higher in HF diet-fed mice than in N diet-fed mice (Figure 2(a)). After berberine treatment, body weight was significantly decreased compared to HF diet-fed mice (on 66^th^,69^th^, 72^th^, and 75^th^ day: *P* < 0.05). Daily food intake was not significantly different between the N and HF diet groups, but daily food intake of the BBR-treated group continued to diverge from the control group in HF diet [*P* < 0.05; Figure 2(b)].

#### 3.1.2. Fat Storage

**Figure 3:**
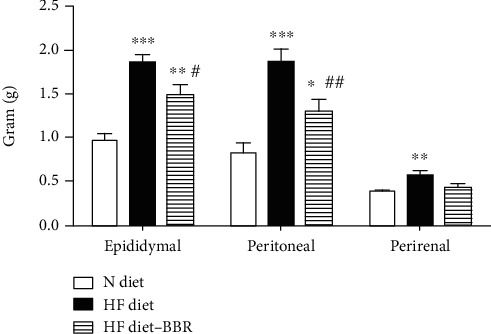
The effects of berberine on body accumulation of the mice. The mass of various adipose tissues (epididymal, perirenal, and peritoneal) was shown in terms of per body weight (kg). Parallel to the body weight change, the weights of regional fat mass were higher in the HF diet group than in the N diet group. ^∗^*P* < 0.05, ^∗∗^*P* < 0.01, ^∗∗∗^*P* < 0.001 N diet group vs. HF diet group. ^#^*P* < 0.05, ^##^*P* < 0.01, HF diet group vs. berberine group.

The effects of BBR on body accumulation were shown in Figure 3. The mass of various adipose tissues (epididymal, perirenal, and peritoneal) was shown in terms of per body weight (kg). Parallel to the body weight change, the weights of regional fat mass were higher in the HF diet group than in the N diet group. Berberine-treated mice significantly reduced the relative epididymal and peritoneal fat mass that was remarkedly reduced in compared to HF diet-fed mice. There were significant differences in epididymal fat mass (*P* < 0.05) and peritoneal fat mass (*P* < 0.01) which were distinguishable among groups.

#### 3.1.3. Biochemical Analysis

**Figure 4:**
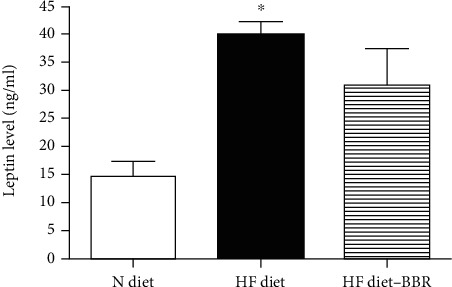
The results of the serum leptin level of the mice. ∗P <0.05, N diet group vs. HF diet group.

*(1) Leptin*. The results of the serum leptin level were shown in Figure 4. The serum leptin level in the HF diet group was significantly higher than that in the N diet group, suggesting the development of hyperleptinemia. In contrast to HF-fed mice (39.8 ± 4.5 ng/ml), those treated with berberine (30.8 ± 13 ng/ml) showed in serum leptin but significant difference was not found among groups.

### 3.2. Experiment 2

#### 3.2.1. Food Intake

**Figure 5:**
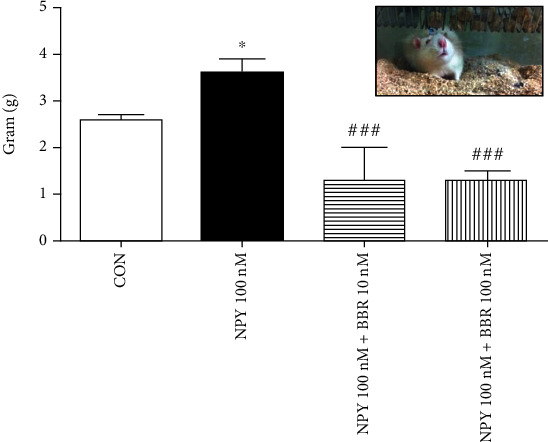
The change of food intake of the rats. ∗P <0.05, CON vs. NPY 100nM. ###*P* < 0.001 BBR group vs. NPY 100nM group.

The change of food intake was shown in Figure 5. Daily food intake was not significantly different between the CON and NPY-treated groups, but with BBR co-injections, the total food consumption during the one hour was significantly different among groups (*P* < 0.001), being 2.6 ± 0.1 g (CON), 3.6 ± 0.3 g (NPY 100 nM), 1.3 ± 0.7 g (BBR 10 nM), and 1.3 ± 0.2 (BBR 100 nM), respectively [*F*_3,16_ = 9.3, *P* < 0.01].

#### 3.2.2. Glucose

**Figure 6:**
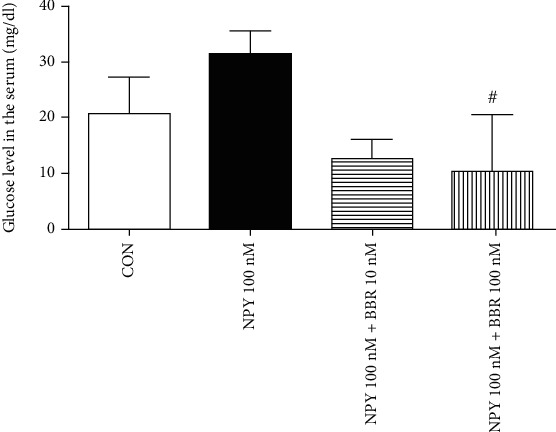
The results of the serum glucose level of rats. #*P* < 0.05 BBR group vs. NPY 100nM group.

The results of the serum glucose level were shown (Figure 6). The serum glucose level in the BBR (100 nM) group was significantly lower than that in the NPY (100 nM) group (*P* < 0.05).

## 4. Discussion

Present study proved that treatment with berberine in the HF diet group reduced food intake, body weight, fat contents, serum leptin, and glucose level. In the rat, ICV injections were accomplished through stereotaxically implanted cannula aimed at the third ventricle. Experiments were carried out in 24-hour fasted rats, and food intake and glucose level were subsequently recorded for 1 hour. ICV injection of NPY increased food intake by 39.3%, compared with the ACSF-injected CON group, but the microinjections of BBR with NPY significantly reduced food intake in rats. The results suggest that BBR improved lipid dysregulation in obesity by controlling the central obesity-related pathway.

Berberine, that is an alkaloid extracted from Rhizoma coptidis, has been used in traditional medicine to treat infection. Recently, some studies have been shown both in vitro and in vivo to have potential use as an antiobesity agent [15–24]. Also, polyphenol intake has benefic role in obese individuals [25]. In the present study, we used a high-fat diet-induced obesity mice model to verify the inhibitory effects of berberine. We also demonstrated that berberine had an effect on central factors known to be involved in the development of obesity. Following BBR-treated mice, we observed a marked decrease in body weight and food intake along with a reduction in the ratio of fat contents to total weight. Furthermore, blood leptin and glucose level of high-fat diet-induced obesity mice were all lowered, showing berberine to be a potential natural that did not produce any physiological changes in normal diet mice. Our data showed that carriers of the berberine-treated mice were more responsive to weight loss in the regulation of abdominal fat distribution, thus raising the question of the mechanism by which berberine affects appetite. One possibility may be via the neuronal system, and this is supported by a recent study demonstrating that berberine improved lipid dysregulation in obesity by controlling the central obesity-related pathway.

Neuropeptide Y is a key peptide affecting adiposity and has been related to obesity risk such as food intake [26] and cardiovascular regulation [27, 28]. Studies have also indicated a pivotal role of NPY in determining body fat distribute via white adipose tissue metabolism [29, 30]. Another study reported that intracerebroventricular administration of neuropeptide Y to normal rats induces a syndrome characterized by obesity, hyperinsulinemia, insulin resistance, and overexpression of the adipose tissue *ob* gene [31]. Clinical study has shown to determine the efficacy and safety of berberine in the treatment of type 2 diabetic patients [32]. Although berberine cannot easily pass the blood-brain barrier, studies have shown that acute administration of berberine intraperitoneally or orally results in an increase of the serotonin level in the brain [33, 34]. Brain serotonin inhibits food intake, while depletion of brain serotonin promotes hyperphagia and weight gain [35].

## 5. Conclusion

From this study, we demonstrate that berberine has an excellent potential as an effective antiobesity agent. However, for more successful therapeutic strategies, we need more in-depth knowledge of the neuronal circuits in which they are working, the downstream targets, and compensatory mechanisms not only in rodent but, critically, also in humans.

In mice, treatment with berberine in the HF diet group reduced the body weight, total food intake, and fat contents to levels equal to the N diet group. In rats, microinjection of berberine in the hypothalamus region reduced food intake and glucose increases. The results suggest that BBR improved lipid dysregulation in obesity by controlling the central obesity-related pathway.

## Figures and Tables

**Table 1:** Composition of the experimental diets (g/kg diet).

Ingredients	Normal diet^1)^	High fat diet^2)^
Casein	200	200
DL-methionine	3	3
Corn starch	150	150
Sucrose	500	345
Cellulose	50	50
Corn oil	50	—
Beer tallow	—	205
Salt mixture	35	35
Vitamin mixture	10	10
Choline bitartrate	2	2
Fat % (calories)	11.7	40.0

^1)^Normal diet: AIN-76A diet #100000 (Dyets Inc., Bethlehem, PA, USA). ^2)^High fat diet: AIN-76 diet #100496 (Dyets Inc., Bethlehem, PA, USA).

## Data Availability

All data used during the study are available in the article and can be solicited from the corresponding author.
