# Corrigendum to “Berberine for Appetite Suppressant and Prevention of Obesity”

**DOI:** 10.1155/bmri/9876059

**Published:** 2025-07-31

H.-J. Park, E.Y. Jung, and I. Shim, “Berberine for Appetite Suppressant and Prevention of Obesity,” *BioMed Research International* 2020 (2020): 3891806, https://doi.org/10.1155/2020/3891806.

In the above article, there was an error in Table 1. The table should show “Beef tallow,” not “Beer tallow.” The corrected table is shown and is listed as Table 1:

We apologize for this error.

## Figures and Tables

**Table 1:** Composition of the experimental diets (g/kg diet).

**Ingredients**	**Normal diet** ^**1)**^	**High-fat diet** ^**2)**^
Casein	200	200
DL-methionine	3	3
Corn starch	150	150
Sucrose	500	345
Cellulose	50	50
Corn oil	50	—
Beef tallow	—	205
Salt mixture	35	35
Vitamin mixture	10	10
Choline bitartrate	2	2
Fat % (calories)	11.7	40.0

^1)^Normal diet: AIN-76A diet #100000 (Dyets Inc., Bethlehem, PA, USA).

^2)^High-fat diet: AIN-76 diet #100496 (Dyets Inc., Bethlehem, PA, USA).

